# Mirror Movements in Acquired Neurological Disorders: A Mini-Review

**DOI:** 10.3389/fneur.2021.736115

**Published:** 2021-09-20

**Authors:** Ping Liu, Yuan Yuan, Ning Zhang, Xiaoyan Liu, Lihua Yu, Benyan Luo

**Affiliations:** ^1^Department of Neurology, the First Affiliated Hospital, Zhejiang University School of Medicine, Hangzhou, China; ^2^Department of Neurology, Pujiang People's Hospital, Jinhua, China

**Keywords:** mirror movements, acquired neurological disorders, transcranial magnetic stimulation, mirror activity, motor evoked potential

## Abstract

Mirror movements (MMs) are specifically defined as involuntary movements occurring on one side of homologous muscles when performing unilateral movements with the contralateral limb. MMs have been considered a kind of soft neurological signs, and the persistence or reappearance of MMs in adults is usually pathologic. In addition to some congenital syndrome, MMs have been also described in age-related neurological diseases including pyramidal system diseases (e.g., stroke, amyotrophic lateral sclerosis) and extrapyramidal disorders (e.g., Parkinson's disease, essential tremor). With the advances in instrumentation and detection means, subtle or subclinical MMs have been deeply studied. Furthermore, the underlying mechanism is also being further elucidated. In this mini-review, we firstly discuss the MM examination means, and then review the literature regarding MMs in individuals with acquired neurological disorders, in order to further understand the pathogenesis of MMs.

## Introduction

Mirror movements (MMs) refer to involuntary movements that appear during voluntary activity in the contralateral homologous muscles. Alongside associated movement and contralateral motor irradiation, MMs are a form of motor overflow phenomena (1–3). However, compared with the other forms, MMs have received the most attention from researchers. Physiological MMs may present during infancy stage in healthy children and persist until around the age of 10 years. They can also be elicited in healthy young and older adults under conditions of severe fatigue, intense physical activity, movements involving large force generation, and proximal muscle use (4, 5). Persistence of involuntary synkinetic mirror movements of the opposite limb is considered pathological.

MMs can be seen in a number of congenital diseases including Klippel-Feil syndrome (6), X-linked Kallman's syndrome (7), and hemiplegic cerebral palsy (8). Cerebral palsy is usually caused by damage that occurs to the immature brain as it develops, most often before birth. Ipsilateral corticospinal tract reorganization is the most accepted theory (9). MMs may also emerge later in life along with neuropsychiatric disorders, such as those associated with lesions in the pyramidal system, that is, stroke (10) and amyotrophic lateral sclerosis (ALS) (11), or extrapyramidal diseases such as Parkinson's disease (PD) (12, 13) and essential tremor (ET) (14). As the result of evolutionary advances in instruments and detection methods, such as surface electromyography (EMG) techniques, transcranial magnetic stimulation (TMS), and functional brain imaging, subtle mirroring or subclinical MMs have been fully revealed and the underlying mechanisms of MMs have been studied comprehensively (2). In addition to cortical origin theories, a subcortical contribution has been proposed. The goal of the current paper was to review the extensive literature regarding MMs in individuals with acquired neurological disorders while acknowledging the involvement of MMs in psychiatric disorders (15).

## Evaluation

### Clinical MMs

Clinical MMs or overt MMs are usually evaluated according to the methods of Woods and Teube (8, 16). Participants are instructed to perform sequential unilateral voluntary motor tasks with either the right or left limb such as finger tapping, fist rotation, finger alternation, opening and closing of the hand, hand pronation-supination, and ankle flexion-extension foot taps. During these activities, patients are asked to rest their inactive arm in their lap and plant their inactive foot lightly on the ground. During the assessment, clinicians can refer to the items on the motor subscale of the Movement Disorder Society-sponsored revision of Unified Parkinson's Disease Rating Scale (MDS-UPDRS) (finger tapping, UPDRS item 3.3; rapid hand opening and closing, item 3.4; rapid pronation-supination movement of hands, item 3.5; and toe tapping, item 3.6) (17, 18).

Motor performance can be videotaped for scoring according to a three-item scale. Evaluation of mirroring severity often includes three aspects: amplitude (range of excursion of the fingers and wrist for hand MMs, or that of the ankle for foot MMs), distribution (extent to which the movements matched those of the limb performing the task), and proportion (fraction of time during which movements were noted). When assessing patients with PD, it is optimal to score MMs in both the “on” and “off” phases (19, 20).

### Electromyographical MMs (e.g., Mirror Activity)

For subjects without clinical evidence of MMs, surface EMG techniques can be used to detect subtle mirroring in the mirror hand (13, 20, 21). Researchers usually use the term mirror activity (MA) to describe the neural concomitant of mirrored EMG activity. Involuntary mirror EMG activity may be recorded when patients keep light background isometric (tonic) muscle contraction in the mirror limb while performing voluntary phasic contractions with the opposite, homologous muscle. Subjects were also usually asked to make a unilateral phasic task (e.g., contraction of abductor pollicis brevis as “brief and brisk” as possible; self-paced sequential finger-thumb oppositions) (11, 21). The most common recording sites include the bilateral first dorsal interosseous (FDI), abductor pollicis brevis (APB), flexor digitorium super ficialis (FDS), and tibialis anterior (TA) muscles.

The mean EMG amplitude was measured in the mirror muscle during the 50 ms after burst onset in the voluntary one and expressed as a percentage of the mean background EMG level in the mirror muscle in a time window of 1 s before voluntary muscle burst onset. Besides, the absolute values of the peak amplitude of the EMG burst in the voluntary muscle and of the mean background EMG level in the mirror one were obtained (21). These surface EMG techniques have been used in different groups of people, such as those with PD (21), ALS (11), and normal adult subjects.

### MM Potential Elicited by TMS

Transcranial magnetic stimulation (TMS) is a safe, non-invasive technique that has been used to obtain important information about cortical function (22). TMS delivers electromagnetic pulses to the cerebral cortex through a magnetic coil. When a single pulse of TMS is applied to an inactive region of the primary motor cortex (M1) with satisfactory intensity, it can depolarize corticospinal neurons and elicit a contraction in the contralateral target muscles. This contraction can be recorded *via* surface EMG and is described as a motor-evoked potential (MEP) (22). While a single contralateral MEP is expected, bilateral MEPs (at similar or slightly shorter latencies) may indicate an active ipsilateral corticospinal tract. The application of TMS to an active motor cortex will initially induce a facilitated response followed by the suppression of tonic activity (i.e., a silent period) (15, 23). Normally, ipsilateral motor-evoked potentials (iMEPs) can be detected in healthy children under the age of 10. From the age of 10 onwards, iMEPs are not detected (24). iMEPs have been reported in patients with congenital mirror movements (CMM) (25), stroke (26–28), and ALS (29).

## Clinical Characteristics of MMs in Different Acquired Neurological Diseases

### MMs in PD

#### Clinical Characteristic

PD is a progressive neurodegenerative disorder characterized by the loss of pigmented cells in the substantia nigra. The clinical features include bradykinesia, rigidity, and/or resting tremor. MMs have been previously reported to occur in 29–95.7% of PD patients (12, 13). The differences in MM prevalence among studies were mainly related to the symptom severity and assessment methods. MMs mainly occur on the less-affected side in patients with asymmetric parkinsonism (Supplementary Video 1). In addition, the MMs had a significant linear correlation with the asymmetry degree of motor deficits. The presence of MMs on the less-affected side were more frequently observed during alternate movements or repetitive flexion/extension movements of the wrist than for finger tapping (17, 30). MM severity was also correlated with the “on” and “off” states such that mirroring was slightly greater when the patients were off medication (21). However, Chatterjee et al. found that MM scores were higher for lower limb tasks in the on phase (12). By applying surface EMG in PD patients without overt MMs, Cincotta found that MA during intended unimanual movements was significantly enhanced compared with age-matched or young healthy volunteers (21). Sharplesa further compared MA in PD patients with and without overt MMs with that in controls (31). They found that MA was enhanced in both PD groups during submaximal contractions, and that the incidence of MA was significantly higher in PD patients with overt MMs (31). A longitudinal assessment showed that MMs persisted beyond 5 years of disease evolution and for at least 2–3 years after the onset of dopaminergic treatment. MMs are known to diminish as PD progresses, so they are considered an early sign of PD.

In PD subjects, dopaminergic therapy has a significant effect on MMs. The better the motor response to dopaminergic drugs, the more obvious the mirroring is in asymmetric idiopathic PD. It has been speculated that the improvement in bradykinesia and rigidity on the less-affected limbs after dopaminergic treatment may facilitate more MMs to occur (32). To support this speculation, we observed that MMs occurred in a PD patient with levodopa-induced dyskinesia (Supplementary Video 2). The effect of dopaminergic drugs has also been studied in patients without overt MMs, using surface EMG of right and left APB contractions (30). In this study, the magnitude of EMG-detected mirroring was slightly greater in the off compared with the on state, although this difference was not significant (19).

#### Pathophysiology

Cortical and subcortical mechanisms have been proposed to explain the MM phenomenon in PD patients. Since MMs mainly present in the early phases of the disease, particularly in individuals with asymmetric motor symptoms, potential underlying mechanisms include disturbed interhemispheric balance of cortical excitability, movement lateralization, and transcallosal inhibition (20).

Several researchers have examined the pathophysiological mechanism of MMs in PD patients *via* surface EMG analyses combined with focal TMS. Cincotta et al. showed that focal TMS of the primary motor cortex (M1) could elicit normal MEPs in the contralateral abductor pollicis brevis (APB), but not in the ipsilateral hand. During both mirror and voluntary movements of one hand, TMS of the contralateral M1 produced a similar, long-lasting silent period (SP), but TMS of the ipsilateral M1 produced a short SP. During either mirror or voluntary APB contraction, paired-pulse TMS elicited a reduction in short-interval intracortical inhibition in the contralateral M1 (33). Furthermore, Li demonstrated that in PD patients with unilateral MMs, the SP in the hand ipsilateral to the one affected by MMs was shorter than that in the unaffected hand and that in controls (34). An ipsilateral SP (iSP) is a TMS-based measure of inhibition between the bilateral M1, likely due to transcallosal inhibitory circuits. The presence of an iSP suggests that MMs in PD may be caused by decreased interhemispheric inhibition, leading to increased motor output from the M1 ipsilateral to the voluntary movements, through crossed corticospinal pathways. Sharples et al. studied MA during maximal and submaximal finger contractions in PD patients and used TMS in a paired pulse paradigm to evaluate interhemispheric inhibition (IHI) of the ipsilateral motor cortex. They found that while ipsilateral motor cortex excitability was the highest in PD patients with overt MMs, IHI did not differ between PD patients and controls. Furthermore, while 5 Hz rTMS to the supplementary motor area (SMA) reduced IHI in PD patients without MMs, it did not affect MA (31). The above findings indicate that decreased IHI may not be the unique contributor to overt MMs in PD. Instead, MMs in this population may be due to the combination of enhanced ipsilateral motor cortex excitability and an earlier onset of EMG activation in the mirror hand.

A functional magnetic resonance imaging (fMRI) study (35) showed that MMs in patients with asymmetrical PD were associated with deactivation of the non-mirroring inhibitory network (dorsolateral prefrontal cortex, presupplementary motor area), as well as overactivation of prokinetic areas (especially the insula). In drug-naive PD patients with only right hemiparkinsonian symptoms, fMRI showed decreased activity in the left putamen and left supplementary motor area but increased activity in the right primary motor cortex, right premotor cortex, left postcentral gyrus, and bilateral cerebellum. The connectivity from the left putamen to cortical motor regions and the cerebellum was decreased, while interactions between the cortical motor regions, cerebellum, and right putamen were increased in this population (36). This suggests that dysfunction of the striatal-cortical circuit might be a subcortical explanation for some motor deficits in PD, such as MMs.

### MMs in Corticobasal Syndrome

Corticobasal syndrome (CBS) is a clinical syndrome presenting with progressive asymmetric bradykinesia, rigidity, and dystonia accompanied by multiple cortical signs. MM is considered a cortical sign of CBS, which can occur independently or accompany with other cortical signs, such as apraxia, alien limb phenomena, cortical sensory loss, and myoclonus. CBS is highly heterogeneous in pathology and is associated with different pathological conditions including corticobasal degeneration, progressive supranuclear palsy, Alzheimer's disease, Creutzfeldt-Jakob's disease, and dementia with Lewy bodies (37). Recently, Paramanandam et al. (38) reported a middle-aged woman with progressive left-hand dystonic posturing and ideomotor apraxia, as well as MMs of upper limbs and stimulus-sensitive myoclonus, initially diagnosed with probable CBS. However, neuropathological examination showed widespread glial cytoplasmic alpha-synuclein accumulation in the corticopontine fibers, pontine gray matter, and oligodendroglia, and the final pathological diagnosis was multiple system atrophy (38). MMs of CBS occur predominantly in the left hand, which is the more affected side (38, 39).

### MMs in Essential Tremor

ET is one of the most common movement disorders. It is characterized by bilateral limb kinetic/postural tremor, with or without tremor in other body parts including the head and lower limbs, as well as vocal tremor. Louis et al. first reported MM phenomena in ET patients in a clinical-epidemiological study, where MMs were present in 32.7% of ET cases (14). However, in another clinical-epidemiological study, the prevalence was as high as 77.7% (40). Although both studies used videotaped neurological examinations to evaluate MMs, the two samples differed substantially in terms of the patient age as well as disease duration and severity. MMs were most common and most severe in ET cases with rest tremor, but there was no correlation with age, tremor duration, or severity, or with MMSE scores. There is a substantial body of evidence to support the idea that some ET patients have an increased risk of developing PD (41). Besides tremor, MMs seem to be another overlapping clinical feature of ET and PD.

At present, no studies have examined the pathophysiological mechanisms of MMs in ET. Whether ET patients with both rest tremor and MMs have a higher chance of progressing to PD remains to be confirmed by longitudinal cohort studies.

### MMs in Amyotrophic Lateral Sclerosis

#### Clinical Characteristic

ALS, also known as Lou Gehrig's disease, is a neurodegenerative disorder characterized by progressive and selective degeneration of both upper and lower motor neurons, usually in an asymmetrical manner. Physical signs of upper motor neuron (UMN) degeneration include hyperreflexia, increased muscle tone with spasticity, and an increased extensor plantar response. However, clinical signs of UMN degeneration are often difficult to elicit in patients with ALS, and there are few accepted and reliable markers for monitoring UMN abnormalities (42, 43).

Overt MMs have been reported in 25%−39% of ALS patients with or without clinical signs of UMN degeneration (11, 44, 45) and are considered to be an early sign of UMN damage in this population. MMs may present in all stages of the disease, and the occurrence of MMs is significantly correlated with scores on the revised ALS functional rating scale. MMs were stronger in patients with greater symptomology (11).

Krampfl identified MMs clinically in 27% and electromyographically in 50% of ALS patients. Using EMG combined with TMS (unilateral stimulation *via* TMS while recording MEPs from the bilateral abductor pollicis brevis muscle simultaneously by means of surface electrodes), iMEPs following TMS were detected in 61% of all ALS patients, and in 47% of patients with suspected ALS without clinical UMN signs (11). This suggests that TMS in conjunction with EMG to record iMEPs is a sensitive method for detecting MMs, and that this approach was superior to mere clinical observation or contralateral EMG recordings.

#### Pathophysiology

The cortical silent period, which mainly represents cortical inhibition, is a period of EMG silence during muscle contraction following a TMS-evoked motor response (46). Wittstock reported that ALS patients with MMs had disturbed transcallosal inhibition (TI), for example, prolongation of latency or loss of the iSP in at least one hemisphere. The involvement of transcallosally projecting intracortical inhibitory output neurons may commence at early stages of the disease. Recently, Wittstock attempted to elucidate the functional and structural alterations of callosal integrity in ALS patients with MMs by means of TMS and diffusion tensor imaging (45). They investigated the iSP as a measure of transcallosal inhibition, and diffusion changes in the corpus callosum and corticospinal tract as a measure of structural integrity. The results showed that ALS patients with MMs had a prolongation of latency or loss of iSP, but no changes in diffusion in the corpus callosum. Thus, functional disturbances of transcallosal pathways may precede microstructural changes in the corpus callosum (47).

### MMs in Stroke

#### Clinical Characteristic

MMs have been reported as a complication of hemiplegic stroke, both in cortical and subcortical structures (internal capsule, basal ganglia, brain stem, etc.) (10, 28, 48–51). MMs are usually observed in non-paretic limbs when patients move the paretic limb and mostly occur in the hands, although they are occasionally present in the leg or foot. The incidence of MMs in stroke patients ranges from 54.8 to 70% (51). This variation may reflect differences in the sensitivity of MM detection methods. Compared with stroke patients without MMs, those with MMs in the unaffected hand exhibit greater motor deficits in the paretic hand, and the magnitude of MMs is correlated with the severity of motor dysfunction (48).

Few studies have examined the time course of MMs in stroke patients. Chieffo showed that MMs presented at the acute phase (4–12 days) in subcortical infarction patients and had decreased significantly at 1 month poststroke (28). Ejaz found that MMs in the non-paretic hand were robust immediately after a first-time stroke (Week 2) but progressively diminished over the following year with a time course that paralleled individuation deficits in the paretic hand (52). Ohtsuka conducted longitudinal follow-ups to assess MMs after right pontine infarction. They found that MMs decreased with time and that this decrease was concomitant with an improvement of motor function in the affected hand (53). However, the first follow-up assessment took place 3 months after the stroke, which was later than in the study by Ejaz (52). The severity of MMs in the unaffected hand is closely related to the motor function in the affected hand. Generally, MMs in the unaffected hand change over time but persist in patients with poor outcomes.

#### Pathophysiology

Functional brain imaging has provided insights regarding the mechanisms underlying MMs in the unaffected hand after an adult-onset stroke. One possible explanation is hyperactivation of the non-lesioned hemisphere after stroke.

Positron emission tomography studies of patients with adult-onset stroke and MMs in the unaffected hand during active movements of the paretic hand have shown a significant increase in regional cerebral blood flow in the unaffected sensorimotor cortex (54). fMRI studies have also reported increased activity in the non-lesioned sensorimotor cortex poststroke (55). Activity in the contralesioned sensorimotor areas might lead to MMs in the non-paretic hand *via* the crossed corticospinal tract. However, Ejaz studied finger recruitment patterns in the non-paretic hand during mirroring using a custom-built ergonomic keyboard and reported that evoked BOLD fMRI responses in S1/M1 were remarkably stable throughout recovery. They found no overactivation in the sensorimotor cortices in either hemispheres. The author speculated that MMs after stroke had a subcortical origin, such as the reticulospinal system (52). Using the H-reflex technique to test the spinal excitability of resting muscles, Caronni observed abductor pollicis brevis and abductor digiti minimi H-reflexes in the paretic hand in stroke patients. This suggests the presence of increased spinal excitability in the muscles of the paralyzed hand, because H-reflexes are rarely recorded in healthy subjects (56).

## Discussion

Mirror movements is a rare physical sign which can be elicited both in pyramidal as well as extrapyramidal disorders. In PD, ALS, and stroke, MMs usually occur in the early stages and may disappear with disease progression. MMs is associated with asymmetry of parkinsonism of PD and also influenced by dopaminergic therapy. MMs were most common and severe in ET cases with rest tremor, indicating those patients may have an increased risk of developing PD. For ALS, MMs have been reported in patients with or without clinical signs of UMN degeneration, and they were more obvious in those with greater motor dysfunction. The value of this mirroring phenomenal to represent an early UMN sign needs further exploration.

From another perspective, MMs can be seen in patients with hyperkinetic and hypokinetic disorders. Huntington's disease (HD) is a representative of hyperkinetic disorder, and its hallmark symptom is the presence of progressive chorea. MMs have been reported in HD, and the degree of MMs in HD was positively correlated with overall motor symptom severity (57). Considering that HD is a hereditary disorder caused by an autosomal dominant mutation, we did not discuss MMs in HD as a separate section, which is a limitation of our text. We found that there were no direct comparison studies of MMs among patients with hyperkinetic or hypokinetic disorders. We speculated that MMs was closely related to the asymmetry of motor dysfunctions in hyperkinetic disorders, while to the severity of motor symptoms in hyperkinetic ones.

Cortical and subcortical mechanisms have been proposed to explain the MM phenomenon both in pyramidal (ALS) as well as extrapyramidal (PD) disorders. In hemiplegic stroke patients, the presentation of MMs both in acute lesions of cortical and subcortical structures (internal capsule, basal ganglia, brain stem, etc.) may further support its different origins. The exact pathophysiology of MMs and their discrepancy among different diseases or stages require further investigation in both healthy and patient populations *via* a range of new emerging technologies.

## Author Contributions

YY and BL developed the search strategy. PL, LY, and NZ conducted the literature search and did the initial screen of articles. PL and XL wrote the manuscript together. All authors contributed to the article and approved the submitted version.

## Funding

This work was supported by the National Natural Science Foundation of China (NO. 81801056).

## Conflict of Interest

The authors declare that the research was conducted in the absence of any commercial or financial relationships that could be construed as a potential conflict of interest.

## Publisher's Note

All claims expressed in this article are solely those of the authors and do not necessarily represent those of their affiliated organizations, or those of the publisher, the editors and the reviewers. Any product that may be evaluated in this article, or claim that may be made by its manufacturer, is not guaranteed or endorsed by the publisher.

## References

[1] Hoy KE Fitzgerald PB Bradshaw JL Armatas CA Georgiou-Karistianis N. Investigating the cortical origins of motor overflow. Brain Res Brain Res Rev. (2004) 46:315–27. 10.1016/j.brainresrev.2004.07.013 15571773

[2] Farmer SF. Mirror movements in neurology. J Neurol Neurosurg Psychiatry. (2005) 76:1330. 10.1136/jnnp.2005.069625 16170069 PMC1739351

[3] Cox BC Cincotta M Espay AJ. Mirror movements in movement disorders: a review. Tremor Other Hyperkinet Mov. (2012) 2. 10.5334/tohm.113 23440079 PMC3569961

[4] Bodwell JA Mahurin RK Waddle S Price R Cramer SC. Age and features of movement influence motor overflow. J Am Geriatr Soc. (2003) 51:1735–9. 10.1046/j.1532-5415.2003.51557.x 14687351

[5] Mayston MJ Harrison LM Stephens JA. A neurophysiological study of mirror movements in adults and children. Ann Neurol. (1999) 45:583–94. 10319880 10.1002/1531-8249(199905)45:5<583::aid-ana6>3.0.co;2-w

[6] Gunderson CH Solitare GB. Mirror movements in patients with the Klippel-Feil syndrome. Neuropathologic observations. Arch Neurol. (1968) 18:675–9. 10.1001/archneur.1968.00470360097009 5652995

[7] Mayston MJ Harrison LM Quinton R Stephens JA Krams M Bouloux PM. Mirror movements in X-linked Kallmann's syndrome. I. A neurophysiological study. Brain. (1997) 120 (Pt 7):1199–216. 10.1093/brain/120.7.1199 9236631

[8] Magne VA Adde L Hoare B Klingels K Simon-Martinez C Mailleux L. Assessment of mirror movements in children and adolescents with unilateral cerebral palsy: reliability of the Woods and Teuber scale. Dev Med Child Neurol. (2021) 63:736–42. 10.1111/dmcn.14806 33469938

[9] Kuo HC Friel KM Gordon AM. Neurophysiological mechanisms and functional impact of mirror movements in children with unilateral spastic cerebral palsy. Dev Med Child Neurol. (2018) 60:155–61. 10.1111/dmcn.13524 28884806 PMC8331099

[10] Nelles G Cramer SC Schaechter JD Kaplan JD Finklestein SP. Quantitative assessment of mirror movements after stroke. Stroke. (1998) 29:1182–7. 10.1161/01.STR.29.6.1182 9626292

[11] Krampfl K Mohammadi B Komissarow L Dengler R Bufler J. Mirror movements and ipsilateral motor evoked potentials in ALS. Amyotroph Lateral Scler Other Motor Neuron Disord. (2004) 5:154–63. 10.1080/14660820410019657 15512904

[12] Chatterjee P Banerjee R Choudhury S Mondal B Kulsum MU Chatterjee K. Mirror movements in Parkinson's disease: An under-appreciated clinical sign. J Neurol Sci. (2016) 366:171–6. 10.1016/j.jns.2016.05.026 27288800

[13] Ottaviani D Tiple D Suppa A Colosimo C Fabbrini G Cincotta M. Mirror movements in patients with Parkinson's disease. Mov Disord. (2008) 23:253–8. 10.1002/mds.21825 17999432

[14] Louis ED Rios E Henchcliffe C. Mirror movements in patients with essential tremor. Mov Disord. (2009) 24:2211–7. 10.1002/mds.22749 19795466 PMC2847261

[15] Hoy KE Georgiou-Karistianis N Laycock R Fitzgerald PB. Using transcranial magnetic stimulation to investigate the cortical origins of motor overflow: a study in schizophrenia and healthy controls. Psychol Med. (2007) 37:583–94. 10.1017/S0033291706009810 17224095

[16] Woods BT Teuber HL. Mirror movements after childhood hemiparesis. Neurology. (1978) 28:1152–7. 10.1212/WNL.28.11.1152 568735

[17] Espay AJ Li JY Johnston L Chen R Lang AE. Mirror movements in parkinsonism: evaluation of a new clinical sign. J Neurol Neurosurg Psychiatry. (2005) 76:1355–8. 10.1136/jnnp.2005.062950 16170075 PMC1739373

[18] Goetz CG Fahn S Martinez-Martin P Poewe W Sampaio C Stebbins GT. Movement disorder society-sponsored revision of the unified parkinson's disease rating scale (MDS-UPDRS): process, format, and clinimetric testing plan. Mov Disord. (2007) 22:41–7. 10.1002/mds.21198 17115387

[19] Cincotta M Borgheresi A Balestrieri F Giovannelli F Ragazzoni A Vanni P. Mechanisms underlying mirror movements in Parkinson's disease: a transcranial magnetic stimulation study. Mov Disord. (2006) 21:1019–25. 10.1002/mds.20850 16547917

[20] Spagnolo F Coppi E Chieffo R Straffi L Fichera M Nuara A. Interhemispheric balance in Parkinson's disease: a transcranial magnetic stimulation study. Brain Stimul. (2013) 6:892–7. 10.1016/j.brs.2013.05.004 23810506

[21] Cincotta M Giovannelli F Borgheresi A Balestrieri F Vanni P Ragazzoni A. Surface electromyography shows increased mirroring in Parkinson's disease patients without overt mirror movements. Mov Disord. (2006) 21:1461–5. 10.1002/mds.20972 16705686

[22] Vucic S Kiernan MC. Transcranial magnetic stimulation for the assessment of neurodegenerative disease. Neurotherapeutics. (2017) 14:91–106. 10.1007/s13311-016-0487-6 27830492 PMC5233629

[23] Thompson PD Day BL Rothwell JC Dressler D Maertens DNA Marsden CD. Further observations on the facilitation of muscle responses to cortical stimulation by voluntary contraction. Electroencephalogr Clin Neurophysiol. (1991) 81:397–402. 10.1016/0168-5597(91)90029-W 1718726

[24] Muller K Kass-Iliyya F Reitz M. Ontogeny of ipsilateral corticospinal projections: a developmental study with transcranial magnetic stimulation. Ann Neurol. (1997) 42:705–11. 10.1002/ana.410420506 9392569

[25] Kim ED Kim GW Won YH Ko MH Seo JH Park SH. Ten-year follow-up of transcranial magnetic stimulation study in a patient with congenital mirror movements: a case report. Ann Rehabil Med. (2019) 43:524–9. 10.5535/arm.2019.43.4.524 31499606 PMC6734025

[26] Fujiwara T Sonoda S Okajima Y Chino N. The relationships between trunk function and the findings of transcranial magnetic stimulation among patients with stroke. J Rehabil Med. (2001) 33:249–55. 10.1080/165019701753236428 11766953

[27] Etoh S Noma T Matsumoto S Kamishita T Shimodozono M Ogata A. Stroke patient with mirror movement of the affected hand due to an ipsilateral motor pathway confirmed by transcranial magnetic stimulation: a case report. Int J Neurosci. (2010) 120:231–5. 10.3109/00207450903404229 20374093

[28] Chieffo R Inuggi A Straffi L Coppi E Gonzalez-Rosa J Spagnolo F. Mapping early changes of cortical motor output after subcortical stroke: a transcranial magnetic stimulation study. Brain Stimul. (2013) 6:322–9. 10.1016/j.brs.2012.06.003 22776700

[29] Krampfl K Petri S Gotz F Mohammadi B Bufler J. Amyotrophic lateral sclerosis (ALS) and mirror movements in a patient with polymicrogyria. Amyotroph Lateral Scler Other Motor Neuron Disord. (2003) 4:266–9. 10.1080/14660820310008515 14753662

[30] Vidal JS Derkinderen P Vidailhet M Thobois S Broussolle E. Mirror movements of the non-affected hand in hemiparkinsonian patients: a reflection of ipsilateral motor overactivity? J Neurol Neurosurg Psychiatry. (2003) 74:1352–3. 10.1136/jnnp.74.9.1352 12933961 PMC1738648

[31] Sharples SA Almeida QJ Kalmar JM. Cortical mechanisms of mirror activation during maximal and submaximal finger contractions in Parkinson's disease. J Parkinson's Dis. (2014). 4:437–52. 10.3233/JPD-130316 24670487

[32] Espay AJ Morgante F Gunraj C Chen R Lang AE. Mirror movements in Parkinson's disease: effect of dopaminergic drugs. J Neurol Neurosurg Psychiatry. (2006) 77:1194–5. 10.1136/jnnp.2005.086892 16980659 PMC2077552

[33] Cincotta M Borgheresi A Ragazzoni A Vanni P Balestrieri F Benvenuti F. Motor control in mirror movements: studies with transcranial magnetic stimulation. Suppl Clin Neurophysiol. (2003) 56:175–80. 10.1016/S1567-424X(09)70219-3 14677392

[34] Li JY Espay AJ Gunraj CA Pal PK Cunic DI Lang AE. Interhemispheric and ipsilateral connections in Parkinson's disease: relation to mirror movements. Mov Disord. (2007) 22:813–21. 10.1002/mds.21386 17290459

[35] Poisson A Ballanger B Metereau E Redoute J Ibarolla D Comte JC. A functional magnetic resonance imaging study of pathophysiological changes responsible for mirror movements in Parkinson's disease. PLoS ONE. (2013) 8:e66910. 10.1371/journal.pone.0066910 23825583 PMC3692538

[36] Wu T Hou Y Hallett M Zhang J Chan P. Lateralization of brain activity pattern during unilateral movement in Parkinson's disease. Hum Brain Mapp. (2015) 36:1878–91. 10.1002/hbm.22743 25644527 PMC6869082

[37] Doi H Tanaka F. [The genetics of corticobasal syndrome]. Brain Nerve. (2013) 65:19–30. 23300100

[38] Paramanandam V Olszewska DA Shakya B Chalissery AJ O'Connell M Farrell M. A 57-year-old woman with progressive left hand clumsiness and falls. Mov Disord Clin Pract. (2019) 6:656–60. 10.1002/mdc3.12825 31745473 PMC6856468

[39] Fisher CM. Alien hand phenomena: a review with the addition of six personal cases. Can J Neurol Sci. (2000) 27:192–203. 10.1017/S0317167100000834 10975531

[40] Louis ED Gillman A. Mirror movements in essential tremor: prevalence and relationship to mini-mental status test scores. Tremor Other Hyperkinet Mov. (2012) 2. 10.5334/tohm.87 23439885 PMC3570028

[41] Tarakad A Jankovic J. Essential tremor and Parkinson's disease: exploring the relationship. Tremor Other Hyperkinet Mov. (2018) 8:589. 10.5334/tohm.441 30643667 PMC6329774

[42] Swash M. Why are upper motor neuron signs difficult to elicit in amyotrophic lateral sclerosis? J Neurol Neurosurg Psychiatry. (2012) 83:659–62. 10.1136/jnnp-2012-302315 22496581

[43] Swash M Burke D Turner MR Grosskreutz J Leigh PN DeCarvalho M. Occasional essay: Upper motor neuron syndrome in amyotrophic lateral sclerosis. J Neurol Neurosurg Psychiatry. (2020) 91:227–34. 10.1136/jnnp-2019-321938 32054724

[44] Wittstock M Wolters A Benecke R. Transcallosal inhibition in amyotrophic lateral sclerosis. Clin Neurophysiol. (2007) 118:301–7. 10.1016/j.clinph.2006.09.026 17140846

[45] Wittstock M Meister S Walter U Benecke R Wolters A. Mirror movements in amyotrophic lateral sclerosis. Amyotroph Lateral Scler. (2011) 12:393–7. 10.3109/17482968.2011.577223 21554031

[46] Chen R Lozano AM Ashby P. Mechanism of the silent period following transcranial magnetic stimulation. Evidence from epidural recordings. Exp Brain Res. (1999) 128:539–42. 10.1007/s002210050878 10541749

[47] Wittstock M Wilde N Grossmann A Kasper E Teipel S. Mirror movements in amyotrophic lateral sclerosis: a combined study using diffusion tensor imaging and transcranial magnetic stimulation. Front Neurol. (2020) 11:164. 10.3389/fneur.2020.00164 32210909 PMC7067896

[48] Kim YH Jang SH Chang Y Byun WM Son S Ahn SH. Bilateral primary sensori-motor cortex activation of post-stroke mirror movements: an fMRI study. Neuroreport. (2003) 14:1329–32. 10.1097/00001756-200307180-00009 12876467

[49] Brainin M Seiser A Matz K. The mirror world of motor inhibition: the alien hand syndrome in chronic stroke. J Neurol Neurosurg Psychiatry. (2008) 79:246–52. 10.1136/jnnp.2007.116046 17578857

[50] Jiang S Zhong D Yan Y Zhu Q Wang C Bai X. Mirror movements induced by hemiballism due to putamen infarction: a case report and literature review. Ann Transl Med. (2020) 8:19. 10.21037/atm.2019.10.86 32055610 PMC6995745

[51] Lee MY Choi JH Park RJ Kwon YH Chang JS Lee J. Clinical characteristics and brain activation patterns of mirror movements in patients with corona radiata infarct. Eur Neurol. (2010) 64:15–20. 10.1159/000313979 20571275

[52] Ejaz N Xu J Branscheidt M Hertler B Schambra H Widmer M. Evidence for a subcortical origin of mirror movements after stroke: a longitudinal study. Brain. (2018) 141:837–47. 10.1093/brain/awx384 29394326 PMC5837497

[53] Ohtsuka H Matsuzawa D Ishii D Shimizu E. Longitudinal follow-up of mirror movements after stroke: a case study. Case Rep Neurol Med. (2015) 2015:354134. 10.1155/2015/354134 26649211 PMC4663000

[54] Auriat AM Neva JL Peters S Ferris JK Boyd LA. A review of transcranial magnetic stimulation and multimodal neuroimaging to characterize post-stroke neuroplasticity. Front Neurol. (2015) 6:226. 10.3389/fneur.2015.00226 26579069 PMC4625082

[55] Wittenberg GF Bastian AJ Dromerick AW Thach WT Powers WJ. Mirror movements complicate interpretation of cerebral activation changes during recovery from subcortical infarction. Neurorehabil Neural Repair. (2000) 14:213–21. 10.1177/154596830001400307 11272478

[56] Caronni A Sciume L Ferpozzi V Blasi V Castellano A Falini A. Mirror movements after stroke suggest facilitation from nonprimary motor cortex: a case presentation. PM R. (2016) 8:479–83. 10.1016/j.pmrj.2015.10.009 26514789

[57] Georgiou-Karistianis N Hoy KE Bradshaw JL Farrow M Chiu E Churchyard A. Motor overflow in Huntington's disease. J Neurol Neurosurg Psychiatry. (2004) 75:904–6. 10.1136/jnnp.2003.016733 15146012 PMC1739065

